# Plasma hsa‐mir‐19b is a potential LevoDopa therapy marker

**DOI:** 10.1111/jcmm.16827

**Published:** 2021-07-30

**Authors:** Aimee Rodica Chis, Alexandra Ioana Moatar, Cristina Dijmarescu, Cecilia Rosca, Ruxandra Julia Vorovenci, Inge Krabbendam, Amalia Dolga, Cristina Bejinar, Catalin Marian, Ioan Ovidiu Sirbu, Mihaela Simu

**Affiliations:** ^1^ Department of Biochemistry “Victor Babes” University of Medicine and Pharmacy Timisoara Romania; ^2^ Center for Complex Networks Science “Victor Babes” University of Medicine and Pharmacy Timisoara Romania; ^3^ Department of Neurology “Victor Babes” University of Medicine and Pharmacy Timisoara Romania; ^4^ Neurology Clinic I Timisoara Emergency County Hospital Timisoara Romania; ^5^ Neurology Clinic SRH Klinikum Karlsbad‐Langensteinbach Karlsbad Germany; ^6^ Department of Molecular Pharmacology Groningen Research Institute of Pharmacy University of Groningen Groningen The Netherlands

**Keywords:** LevoDopa, miR‐19b, Parkinson's disease

## Abstract

Parkinson's disease (PD) is the second most common neurodegenerative disorder among the elderly, the diagnostic and prognostic of which is based mostly on clinical signs. LevoDopa replacement is the gold standard therapy for PD, as it ameliorates the motor symptoms. However, it does not affect the progression of the disease and its long‐term use triggers severe complications. There are no bona fide biomarkers for monitoring the patients’ response to LevoDopa and predicting the efficacy of levodopa treatment. Here, we have combined qPCR microRNA array screening with analysis of validated miRs in naïve versus Levodopa‐treated PD patients. We have identified plasma miR‐19b as a possible biomarker for LevoDopa therapy and validated this result in human differentiated dopaminergic neurons exposed to LevoDopa. In silico analysis suggests that the LevoDopa‐induced miR‐19b regulates ubiquitin‐mediated proteolysis.

## 1 INTRODUCTION

Parkinson's disease (PD) is the second most common motor neurodegenerative disorder among the elderly. PD is characterized by the progressive loss of dopaminergic neurons in the substantia nigra pars compacta.^1^ Due to the lack of specific and sensitive tests or biomarkers, both the diagnostic and the prognostic of PD are based entirely on clinical features, such as the type and severity of symptoms, age, gender or response to therapy.^2^ The most commonly used pharmacological treatment for PD aims to ameliorate the motor symptoms by compensating for the dopamine deficit. This is mainly done through replacement therapy (LevoDopa) with inhibition of Monoaminoxidase (MAO) and Catechol O‐methyltransferases (COMT) activity in the periphery (carbidopa, benserazide).^3^ Long‐term LevoDopa usage associates almost invariably with additional motor side effects and cognitive and neuro‐psychiatric events, while it does not affect or change the natural progression of cell degeneration towards non‐dopaminergic neural circuits.^4^ LevoDopa's altered metabolism in PD patients governs the deleterious feedbacks between the main pathogenic axes, including mitochondrial dysfunction, toxic metabolites, proteasome alteration and alpha‐synuclein aggregation, thus turning the PD cure into a curse in disguise.^5^

MicroRNAs (miRs) are a class of endogenous, small non‐coding RNAs able to post‐transcriptionally modulate gene expression by binding to complementary sequences in target messenger RNAs (mRNAs). One single miR may alter the stability of hundreds of targets, while several miRs may cooperatively interact with one single mRNA and thus regulate the expression of over one‐third of the coding genes in humans.^6^ Due to their outstanding stability in various body fluids, miRs are ideal biomarker candidates in various diseases, including PD.^7^ Interestingly, in PD patients, most of the miRs were shown to be deregulated, with decreased levels in total blood, serum, and white blood cells, while most of the plasma miRs are up‐regulated.^8^, ^9^, ^10^, ^11^, ^12^, ^13^, ^14^, ^15^, ^16^, ^17^, ^18^, ^19^, ^20^, ^21^, ^22^, ^23^, ^24^ However, there is a large discrepancy among these results, presumably due to several differences in their experimental setups. Moreover, many patients are treated with LevoDopa for several years, which could also affect the miRs levels. To shed light on these discrepancies, we aimed to investigate the levels of miRs in the plasma of LevoDopa‐treated PD patients.

In our study, we made use of the sensitivity and specificity of the qRT‐PCR technique to quantify in a two‐step (discovery and validation) approach the mature miR species that are present in the plasma of LevoDopa‐treated PD patients. Analysis of the validated miRs in naïve vs. LevoDopa‐treated PD patients identified plasma miR‐19b as a sensitive biomarker for LevoDopa therapy, a result further validated in mouse hippocampi and human differentiated dopaminergic neurons exposed to LevoDopa.

## 2 MATERIAL AND METHODS

The present study has been performed in concordance with the Declaration of Helsinki Code of Ethics and has been reviewed and approved by the local institutional ethics review board.

All Discovery‐ and Validation lots patients have been recruited through the Neurology Clinic of the County Hospital Timisoara and have signed informed consent. The diagnosis of PD was made following International Parkinson and Movement Disorder Society clinical diagnostic criteria for PD.^25^ Exclusion criteria included cognitive impairment, inability to sign the informed consent, an associated diagnostic of cancer and/or autoimmune disease, and a recent history of head or spinal cord trauma. Control patients are individuals that have addressed the Neurology Clinic and for whom, after clinical and paraclinical examination, a neurological pathology has been excluded.

The naive (and the corresponding post LevoDopa therapy) plasma samples have been provided by the Harvard NeuroDiscovery Center (Center for Neurological Diseases, Brigham & Women's Hospital) and the Neurology Clinic of the County Hospital Timisoara. The demographics and clinical characteristics of the discovery lot, the validation lot, and the post‐therapy lot are presented in Table 1.

**TABLE 1:** Demographics of the discovery, validation and naive lots

	Discovery lot		Validation lot		Naive lot
	Patients	Controls	Patients	Controls	Harvard
No.	10	10	66	29	10
Gender ratio	6/4	6/4	35/31	14/15	7/3
Median age (standard deviation)	65.9 (6.1)	68.1 (6.4)	68 (9.1)	63.5 (9.1)	67.2 (6.6)
Average Y&H stage (standard deviation)	1.9 (0.70)	‐	2.5 (0.8)	‐	‐

Peripheral blood was collected between 9 AM and 12 AM in EDTA‐coated vacutainers by puncture of the cubital vein; next, the blood was refrigerated at 4°C, processed by centrifugation within the next 2 h, and the plasma fraction stored at −80°C until further use. Plasma samples with signs of hemolysis, turbidity, hyperlipidemia or hyperbilirubinemia have been discarded. After thawing, all plasma samples were again centrifuged for 5 min at 1500 g and 4°C to eliminate precipitates and cell debris contaminants. Total RNA was extracted from 200 μl of plasma (100 μl in case of Harvard lot) using the miRNeasy Serum/Plasma kit (Qiagen), with *Caenorhabditis* *elegans* miR‐39 miRNA mimic as a spike‐in control for external normalization.

### 2.1 LevoDopa treatment of mice

The experiments involved two lots (experimental and control) of six one‐year‐old wild‐type mice (three males, three females) of mixed genetic background, housed in Udel^®^ polysulphone cages, on a 12‐h light‐dark cycle and fed ad libitum. L‐DOPA (Sigma) and Benserazide hydrochloride (Sigma) solutions were prepared freshly (30 min before injections) in physiological saline and administered for 5 days, by two 12‐h spaced intraperitoneal injections at 20 mg/kg and 12 mg/kg, respectively. The control lot was injected using the same protocol with equal amounts of Benserazide hydrochloride. The mice were sacrificed on the 6th day, and the hippocampi dissected and stored in RNAlater at −80°C. Total RNA was extracted using the miRVANA kit according to the manufacturer's indications and stored at −80°C until further use.

### 2.2 Dopaminergic cells culture

LUHMES cell differentiation to dopaminergic neural cells was performed as previously described.^26^ Briefly, LUHMES cells were cultured for 24 h in a Dulbecco's modified Eagle's medium (DMEM)/F12 with 1% N2‐supplement and 0.04 μg/ml basic fibroblast growth factor then differentiated for 5 days in DMEM/F12 with 1% N2‐supplement, 1 μg/ml tetracycline, 0.49 mg/ml dibutyryl cyclic AMP and 2 ng/ml glial cell‐derived neurotrophic factor. Differentiated dopaminergic cells were exposed for 24 h to three different concentrations of LevoDopa (10 µM, 20 µM and 50 µM) and then washed with 1x PBS, collected and the pellet frozen at −80°C until further use. Total RNA was extracted using the miRVana kit according to the manufacturer's indications and stored at −80°C.

### 2.3 miRNA and target genes quantification

For discovery lot samples, reverse transcription by miScript II RT Kit, Qiagen (with identical RNA inputs) followed by qRT‐PCR quantification of 1008 unique human miRNAs on a Human miRNome miScript miRNA PCR Array, Qiagen was performed according to the manufacturer's protocols. MiRs showing expression in all samples were used for data analysis by ∆∆CT method of relative quantification with normalization to *C. elegans* miR‐39 miRNA spike‐in.^27^ Validation step and LevoDopa‐induced miR data set analysis.

For the validation lot, the naïve/post‐therapy plasma samples, and the LUHMES cells experiments, reverse transcription was performed using the TaqMan^®^ MicroRNA Reverse Transcription Kit (Thermo Fisher) followed by individual qRT‐PCR quantification using TaqMan™ MicroRNA Assays (Thermo Fisher); all qRT‐PCR reactions were performed in triplicate. For plasma miRs, fold changes were calculated by ∆∆CT method of relative quantification with both exogenous (to *C. elegans* miR‐39 miRNA spike‐in) and endogenous (hsa‐miR‐15b and hsa‐miR‐17) normalization. The two endogenous controls show Ct values that do not differ between experimental and control samples (*p *> 0.05), are expressed at levels comparable to those of our 5 target genes and display coefficients of variation below 15%. Of note, miR‐15b has been highlighted as an endogenous control with very low overall variance, while miR‐17 has been reported as one of the most stable and consistent miR expressed across 13 normal tissues.^26^, ^27^

For mouse hippocampi experiments, reverse transcription was performed using the TaqMan^®^ MicroRNA Reverse Transcription Kit (Thermo Fisher) followed by individual qRT‐PCR quantification using TaqMan™ MicroRNA Assays (Thermo Fisher). All qRT‐PCR reactions were performed in triplicate. The fold changes were calculated by ∆∆CT method of relative quantification using RNU‐44 for normalization.

For LUHMEs cells/dopaminergic neurons microRNA experiments, reverse transcription was performed using the TaqMan^®^ MicroRNA Reverse Transcription Kit (Thermo Fisher) followed by individual qRT‐PCR quantification using TaqMan™ MicroRNA Assays (Thermo Fisher). For target genes experiments, reverse transcription was performed using the High‐Capacity cDNA Reverse Transcription Kit followed by individual qRT‐PCR quantification using inventoried TaqMan™ Assays (Thermo Fisher). All qRT‐PCR reactions were performed in triplicate. The fold changes were calculated by ∆∆CT method of relative quantification using hsa‐RNU‐44 (for miR calculations) and hsa‐Gapdh (for mRNAs calculations) for normalization.

### 2.4 Statistics

In the discovery lot, differentially expressed miRs were identified by two tails, heteroscedastic Student's *t*‐test and ranked according to their corrected *p*‐values (*p *= 0.05 as cut‐off).

In the validation lot, after checking the normalized Ct values for normality of distribution, we evaluated the differential miR expression's statistical significance between patient and control groups using either a 2‐tailed *t*‐test with Welch correction (for normal distribution) or a 2‐tailed Mann–Whitney *U* test for non‐normal distribution (statistical cut‐off: 0.05).

The differential miR expression's statistical significance between naïve and post‐therapy patients’ groups was assessed using the 2‐tailed Wilcoxon signed‐ranks test (*p *< 0.05) for the Harvard lot.

The statistical significance of the differentially expressed miR in the cultured dopaminergic neural cells exposed to LevoDopa was calculated using a two‐way anova test with Dunnett correction. The statistical significance of the differentially expressed target genes in the cultured dopaminergic neural cells exposed to LevoDopa was calculated using a two‐way, unpaired, heteroscedastic *t*‐test with Welch's correction (*p *< 0.05).

The statistical significance of the differentially expressed miR in the mouse hippocampi challenged with LevoDopa was calculated using a two‐way, heteroscedastic, unpaired *t*‐test (*p *< 0.05).

All statistical calculations were performed using Prism 8 for MacOS, version 8.3.0.

The diagnostic test parameters for the three miRs were estimated using the MedCalc online software (https://www.medcalc.org/calc/diagnostic_test.php ).

### 2.5 Bioinformatics analysis

Target predictions (against 3′UTR, 5′UTR and CDS regions) for miR‐19b were computed using the miRWalk 3.0 machine learning algorithm, with a *p *≤ 0.05 (Bonferroni adjusted) as a cut‐off. The miR‐19b targets were cross‐referenced against the differentially expressed genes retrieved after Geo2R analysis (Benjamini and Hochberg adjusted false discovery rate, FDR <0.05) of GSE55096 data set representing the TRAP analysis of striatal neurons’ response to LevoDopa therapy in a 6‐hydroxydopamine (6‐OHDA) mouse Parkinson's disease model.^28^ The miR‐19b targets of the differentially expressed genes were subjected to Gene Ontology analysis on the DAVID platform using adjusted FDR of 0.05 as cut‐off.^29^

## 3 RESULTS

### 3.1 MicroRNA profiling

Out of the 1008 miRs profiled in the discovery group, only 15 were expressed in all plasma samples and exhibited corrected *p*‐values below the 0.05 threshold, with fold changes (FC) varying between 1.5 and 5.1 (Table 2).

**TABLE 2:** MiRs differentially expressed in plasma of PD patients versus controls (discovery lot)

miR	Mapping	FC	*p* Value
hsa‐miR‐19b	chr13: 91351192‐91351278 [+] chrX: 134169671‐134169766 [−]	5.10	0.0412
hsa‐miR‐19a	chr13: 91350891‐91350972 [+]	4.45	0.039
hsa‐miR‐16	chr13: 50048973‐50049061 [−]	3.83	0.012
hsa‐miR‐92a	chr13: 91351314‐91351391 [+]	3.73	0.021
hsa‐miR‐195	chr17: 7017615‐7017701 [−]	3.67	0.019
hsa‐miR‐93*	chr7: 100093768‐100093847 [−]	3.64	0.037
hsa‐miR‐363	chrX: 134169378‐134169452 [−]	3.27	0.019
hsa‐let‐7b	chr22: 46113686‐46113768 [+]	3.10	0.049
hsa‐let‐7e	chr19: 51692786‐51692864 [+]	3.00	0.033
hsa‐miR‐454	chr17: 59137758‐59137872 [−]	2.99	0.023
hsa‐let‐7c	chr21: 16539828‐16539911 [+]	2.74	0.038
hsa‐miR‐16‐2*	chr3: 160404745‐160404825 [+]	2.53	0.016
hsa‐miR‐576‐5p	chr4: 109488698‐109488795 [+]	2.30	0.010
hsa‐miR‐941	chr20: 63919449‐63919520 [+] chr20: 63919505‐63919576 [+] chr20: 63919561‐63919632 [+] chr20: 63919756‐63919827 [+] chr20: 63919868‐63919939 [+]	1.70	0.018
hsa‐miR‐1976	chr1: 26554542‐26554593 [+]	1.51	0.0435

^*^ Belongs to the name of the microRNA.

### 3.2 MicroRNA validation

The differential expression of five of the discovery miRs (miR‐16, miR‐19b, miR‐19a, miR‐92a and miR‐195) was further validated in an additional set of 66 treated PD patients and 29 controls using TaqMan assays. Similar to the discovery lot, all five discovery miRs were up‐regulated in PD patients, with fold changes between 1.31 and 1.72 (Table 3). However, after gender stratification, only miR‐19a, miR‐19b and miR‐195 were found differentially expressed in both male and female patients (miR‐16 and miR‐92a are significantly expressed only in males), thus being further taken into consideration as putative *bona fide* PD biomarkers. There were significant differences in miR‐16 and miR‐92a plasma levels of control males versus control females, while miR‐19a, miR‐19b and miR‐92a are significantly lower in the plasma of PD male patients compared to PD female patients (Table S1).

**TABLE 3:** MiRs differentially expressed in plasma of PD patients versus controls (validation lot)

Fold change (*p*‐Value)	M + F	M	F
hsa‐miR‐16	1.31 (0.026^a^)	1.72 (0.003^a^)	1.31 (0.944^b^)
hsa‐miR‐19b	1.50 (0.001^b^)	1,52 (0.001^b^)	1.50 (0.041^b^)
hsa‐miR‐19a	1.52 (0.001^b^)	1.64 (0.002^a^)	1.52 (0.042^b^)
hsa‐miR‐92a	1.55 (0.009^b^)	1.88 (0.009^b^)	1.55 (0.114^b^)
hsa‐miR‐195	1.72 (0.0008^a^)	1.91 (0.0075^a^)	1.72 (0.043^a^)

Abbreviations: F, females; M, males.

^a^ Two‐tailed Mann–Whitney test.

^b^ Two‐tailed Welch's *t*‐test.

Next, we asked whether the normalized Ct of the five miRs correlate with the age of the probands and the Hoehn–Yahr (H‐Y) stage of Parkinson's disease. Two‐tailed parametric Pearson correlation analysis of PD data indicates that age and H&Y stage do not correlate with, and most probably do not influence the normalized Ct values of any of the validated miRs (Table S2).

### 3.3 Mir‐19a, miR‐19b and miR‐195 as diagnostic biomarkers

**FIGURE 1:**
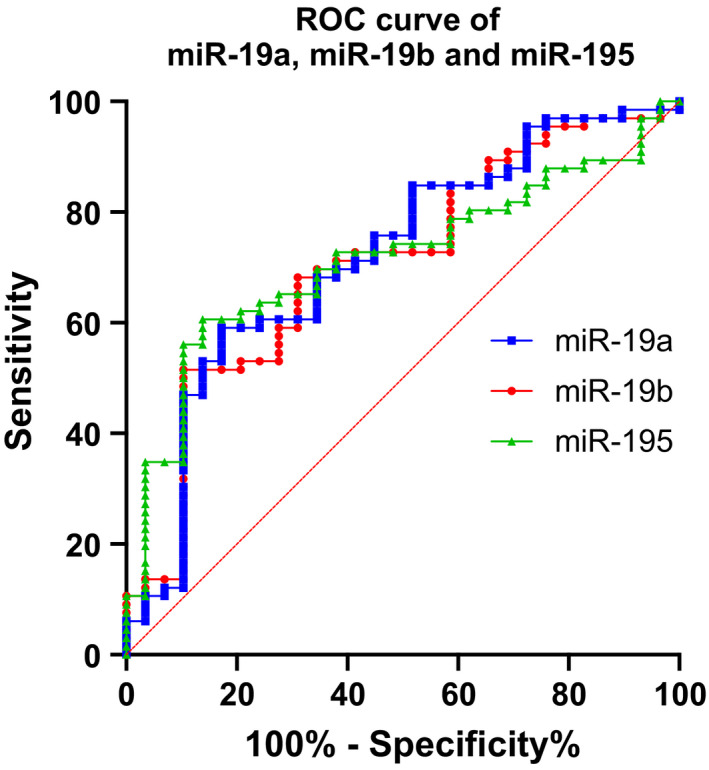
ROC plots of miR‐19a, miR‐19b and miR‐195 in PD patients compared to controls

To evaluate the validated miRs’ ability to discriminate between PD and controls, we performed ROC analyses for all five miRs (Table S3 and Figure 1). This analysis revealed that the five miRs exhibit relatively modest performances, with miR‐19a, miR‐19b and miR‐195 having the highest areas under the curve and the lowest *p* values.

### 3.4 MicroRNA responders to LevoDopa

**FIGURE 2:**
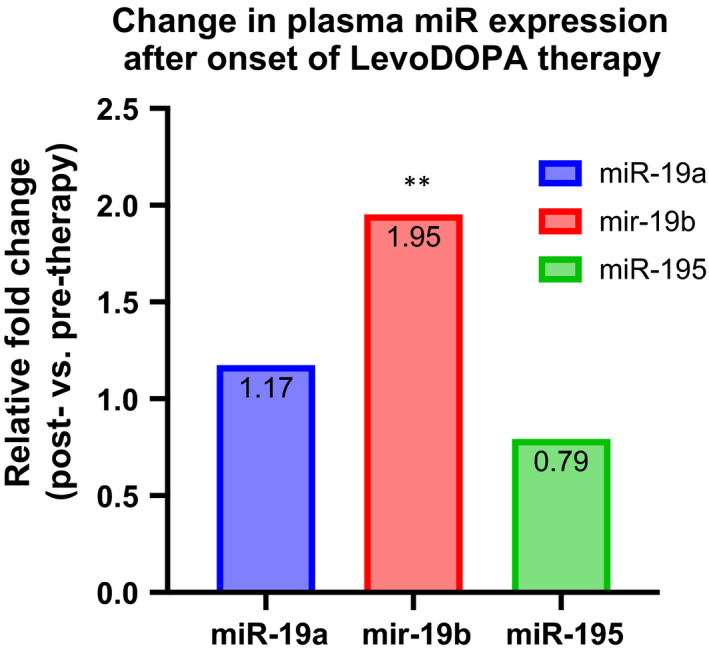
miR‐19a, miR‐19b and miR‐19b expression levels in PD patients’ plasma after onset of substitution therapy versus before onset of substitution therapy

Since all the PD patients enrolled in the validation lot were under replacement/combined therapy, which, as reported, might associate changes in circulating miR levels, we have further compared the miR‐195, miR‐19a and miR‐19b plasma levels in 10 patients (Harvard Neurodiscovery Center) before and after the onset of anti‐Parkinson medication.^12^, ^30^ A Wilcoxon matched‐pairs signed‐ranks test analysis showed a significant change in treated patients for miR‐19b (*p *= 0.0059) but not for miR‐19a and (*p *= 0.9218) and miR‐195 (*p *= 0.1934). Our data indicate a possible role of miR‐19a and mir‐195 in PD biology, while miR‐19b could be considered as a therapy‐responder (Figure 2).

**FIGURE 3:**
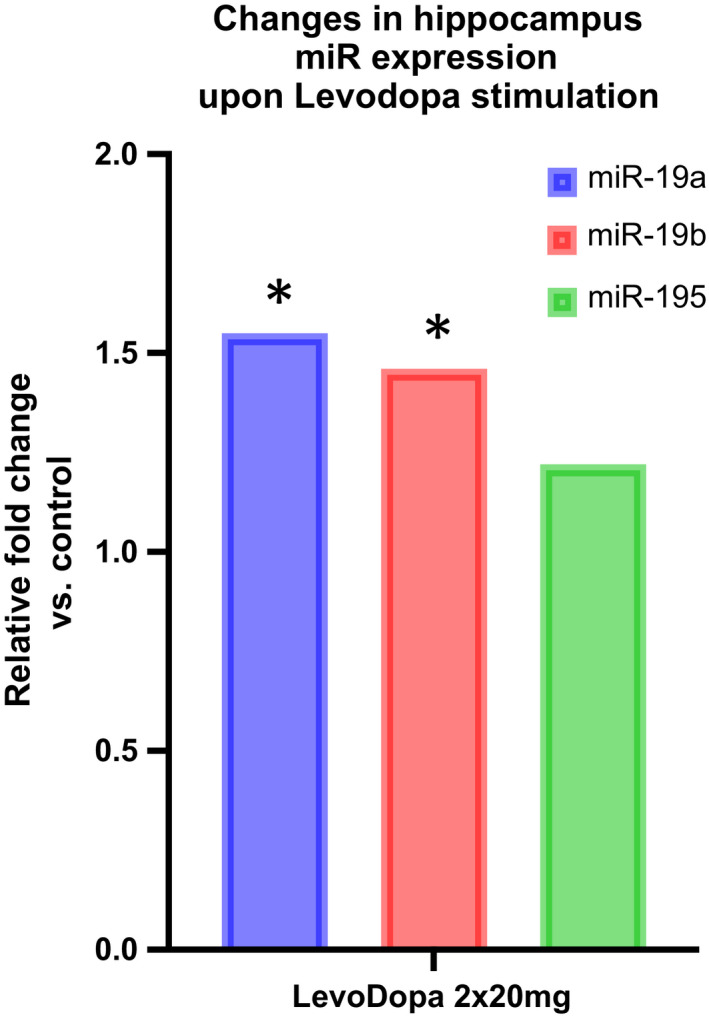
miR‐19a, mir‐19b and miR‐195 expression levels in mouse hippocampi exposed to LevoDopa. *p < 0.05; (unpaired, heteroscedastic Student's *t*‐test)

**FIGURE 4:**
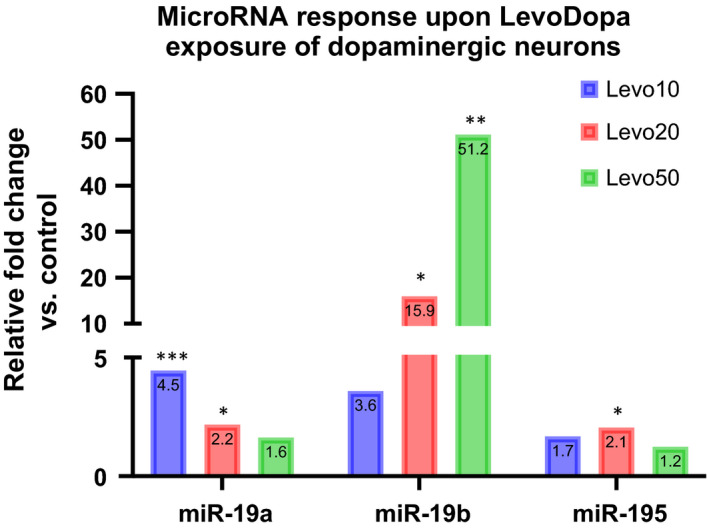
miR‐19a, mir‐19b and miR‐195 expression levels in cultured dopaminergic neurons exposed to LevoDopa. *p < 0.05; **p < 0.01; ***p < 0.005 (two‐way anova test with Dunnett correction)

To further substantiate the possible role of LevoDopa in modulating miR‐195/miR‐19a/miR‐19b expression in tissues relevant for PD pathology, we evaluated their expression in the hippocampi of one‐year‐old, wild type, mixed background mice subjected to five‐day LevoDopa stimulation. The expression of all three miRs increased, however, only miR‐19a and miR‐19b changes were statistically significant (Figure 3). Next, we asked whether the change in miR expression could be specifically reproduced in dopaminergic neurons and monitored the response of cultured LUHMES‐derived dopaminergic neurons to three different levels of LevoDopa. Interestingly, miR‐19a and miR‐195 responded by gradually decreasing their expression upon augmentation of LevoDopa concentration, while miR‐19b showed a dose‐dependent upregulation of expression (Table S4 and Figure 4).

### 3.5 MicroRNA targets

miR‐19b has been proposed as a diagnostic and/or prognostic marker in a wide array of pathologies, from adenoviral and bacterial infections to heart failure, cancer and PD. Mature miR‐19b is exceptionally well conserved in vertebrates (Figure S1), and experiments in mice and rats provided important mechanistic pathogenic details of its involvement in these pathologies.

To gain insight into the possible biological role of miR‐19b upregulation upon LevoDopa exposure, we retrieved and analysed the published transcriptome data of two LevoDopa regimen supplementation experiments in a mouse model of 6‐hydroxydopamine (6‐HODA)‐induced striatal dopamine depletion.^28^ Geo2R analysis (false discovery rate <0.05, Benjamini and Hochberg adjusted) of hemiparkinsonian conditions on LevoDopa therapy versus chronic saline identified 5722 (high LevoDopa regimen) and 2999 (low Levodopa regimen) unique differentially expressed genes (DEG), of which 651 (11.38%) and 378 (12.61%), respectively, are putative 5′‐UTR/CDS/3′‐UTR miR‐19b targets, as predicted using miRwalk3.0 machine learning algorithms.^31^ KEGG Pathway enrichment analysis (FDR < 0.05) of the miR‐19b DEG targets using the DAVID 6.8 platform (https://david.ncifcrf.gov/) identified Ubiquitin‐mediated proteolysis (UDP) pathway as possibly altered by miR‐19b deregulation during LevoDopa supplementation (Table 4 and Figure S2).^29^ Of note, out of the 16 UDP target genes (FBXW8, CUL3, CUL2, UBE2D3, HUWE1, WWP1, KEAP1, NEDD4L, CBL, UBE2J2, UBE2R2, NEDD4, UBR5, BIRC6, TRIM37, ANAPC1), UBE2D3 and TRIM37 have already been validated as miR‐19b targets (MIRT505252 and MIRT500676, respectively) (File S1). CUL3, UBE2D3 and WWP1 have been indexed as miR‐19b targets in miRDB (target scores 74, 87 and 80, respectively), while UBE2D3 (TargetScan probability of conserved targeting: 0.29) and WWP1 (TargetScan probability of conserved targeting: 0.58) have conserved miR‐19b binding sites.

**TABLE 4:** Gene ontology analysis of the miR‐19 targets within the GSE55096 dataset

Term	Count	%	*p*	Fold Enrichment	Bonferroni	Benjamini	FDR
hsa04120: Ubiquitin‐mediated proteolysis	16	2.46	1.53E‐04	3.138	3.46E‐02	3.52E‐02	3.49E‐02
mmu04120: Ubiquitin‐mediated proteolysis	16	2.46	7.04E‐05	3.372	1.58E‐02	1.59E‐02	1.58E‐02

Next, we analysed 22 Parkinson‐related transcriptome data deposited in the Gene Expression Omnibus (GEO) for changes (Geo2R analysis, adjusted FDR < 0.05) in the expression levels of the 16 UDP miR‐19b‐target genes: GSE110716 (fibroblasts, iPSCs, and neurons), GSE68719 (brain tissue), GSE43490 (dorsal nucleus vagus, substantia nigra, locus coeruleus), GSE28894 (frontal cortex, cerebellum, striatum, medulla oblongata), GSE19587 (dorsal motor nucleus of vagus, inferior olivary nucleus), GSE20333 (brain), GSE20295 (prefrontal area, putamen, substantia nigra), GSE20146 (globus pallidus), GSE20141 (substantia nigra pars compacta), GSE6613 (whole blood), GSE72267 (blood), GSE20153 (lymphoblasts) and GSE22491 (peripheral blood mononuclear cells, PBMCs). To our surprise, except for GSE22491 (PBMCs from patients with LRRK2 mutations), none of the GEO data sets survived the Geo2R analysis, adding yet another layer to the well‐known lack of consensus between Parkinson transcriptomes. However, with two exceptions (GSE43490 substantia nigra and locus coeruleus), all data sets (File S2) are consistent in showing decreased expression of ANAPC1 (72.7% of the datasets), UBE2D3 (68.2%), CUL2, CUL3 and KEAP1 (63.6%), while FBXW8 and UBE2J2 are downregulated in only 22.7% of the data sets analysed.

**FIGURE 5:**
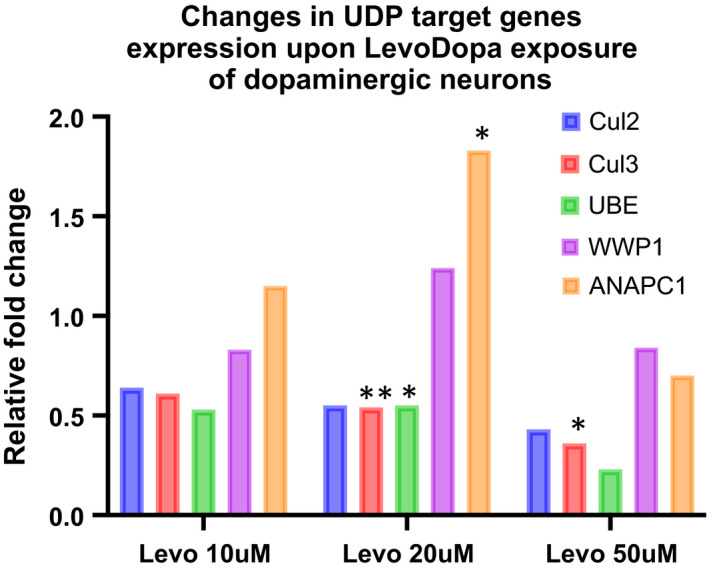
Altered UDP gene expression in cultured dopaminergic neurons exposed to LevoDopa. *p < 0.05; (unpaired, heteroscedastic Student's *t*‐test)

Next, we asked whether the predicted transcriptional impact on UDP pathway genes could be reproduced in our LUHMES‐derived dopaminergic neurons model and quantified the expression of five of the UDP target genes found most consistently downregulated in our GEO data analysis. We found that of Cul2, Cul3 and UBE2D3 are all downregulated, while WWP1 and ANAPC1 expression levels are not changed or even increased in LUHMES‐derived dopaminergic neurons (Figure 5).

Overall, these data indicate a consistent and concordant downregulation of target UDP‐genes transcripts in tissues harvested from PD patients under replacement therapy, mice hippocampi and cultured dopaminergic neural cells challenged with LevoDopa, which might translate into an altered proteasome activity mediated by the LevoDopa‐induced augmentation of miR‐19b.

## 4 DISCUSSION

There is a bewildering lack of consensus between the circulating miRs expression reports, regardless of their origin: plasma, serum or PBMCs of Parkinson's patients^32^; this might reflect not only differences in tissue source, cohort sizes, experimental design (from RNA isolation to validation) but also, in the light of our data, differential individual responses to different LevoDopa therapy dosages.^33^ However, one should also remember there are considerable quantitative and qualitative differences between the miRs’ expression levels in plasma, serum, and white blood cells (WBCs), with high serum levels of miRs rather reflecting the release of miRs associated with the coagulation.^20^, ^34^ From this perspective, one might argue that plasma miRs better reflect the real repertoire of PD‐associated circulating miRs.

Most of the studies that investigated the levels of miRs in serum (including exosomes), PBMCs, and cerebrospinal fluid showed a decreased levels of miR‐19b in PD patients compared to control subjects^8^, ^13^, ^35^, ^36^, ^37^; Marques et al. (2017) could not find any significant change, while Burgos et al. (2014) described statistically significant upregulations of miR‐19b in PD patients.^14^, ^38^ Furthermore, analysis of plasma miRs showed increased levels of miR‐19b in Parkinson's patients versus healthy controls.^39^ Interestingly, global analysis of plasma exosomes did not identify differences in miR‐19b expression, thus excluding exosomes as possible contributors to miR‐19b PD‐associated changes.^40^ It is also worth noting that with very few exceptions, the brain‐enriched miRs investigated so far show increased plasma levels in PD patients, suggesting that the actual miR source might influence the plasma levels in pathological conditions.^15^, ^24^

Very few studies investigated the miR response to LevoDopa therapy in Parkinson's disease, all using a targeted approach and focusing on miR expression in peripheral blood cells. However, the results show the same disconcerting lack of consensus: Alieva et al. described a significant increase of miR‐7, miR‐9‐3p, miR‐9‐5p, miR‐129 and miR‐132, Serafin et al. showed overexpression of miR‐103a, miR‐30b, and miR‐29a, while Caggiu et al. found an upregulation of miR‐155 and downregulation of miR‐146 in the PBMC of LevoDopa‐treated Parkinson's patients.^12^, ^30^, ^41^ Of note, except for miR‐146, all miRs analysed are up‐regulated, including the brain‐enriched miR‐7 and miR‐132.

Our data are too preliminary to support a role for miR‐19b in predicting PD onset in individuals, regardless of their sex and age, an aspect worth exploring in much larger cohorts of probands, which allow proper stratification. Furthermore, we found no correlation between any of the five plasma miRs and the age or H&Y stage of PD patients. Male gender has been highlighted as one of the risk factors for PD development. This aspect seems to be mediated by SRY expression in dopaminergic striatal neurons and strongly reflected at both clinical and molecular/transcriptome levels.^42^, ^43^, ^44^ Interestingly, plasma miR‐16 gender‐dependent differential expression is lost in PD patients, miR‐92a retains its gender‐biased expression, while miR‐19a and miR‐19b are gender‐biased only in PD. Of note, miR‐19a and miR‐19b are considered ‘male‐specific’ miRs, while miR‐195 is rather female‐specific.^45^

An aspect worth investigating would be the mechanism leading to mature miR upregulation upon LevoDopa exposure. While the human plasma and mouse hippocampi show concordant changes in mature miR‐19b and miR‐19a levels, consistent with a common, transcriptional regulation of the miR‐17~92 cluster, the cultured dopaminergic neurons’ response diverges as the intensity of LevoDopa exposure increases, suggesting that different regulatory events might also be at work. Given that miR‐19b is less accessible to Drosha processing from pri‐miR‐17~92 than miR‐19a, it is tempting to speculate that LevoDopa post‐transcriptionally alters the miR expression ratios within this cluster.^46^ Whether this LevoDopa‐induced change in miR‐19a/miR‐19b ratio scenario also applies to the miR‐106a‐303 cluster (the chromosome X source of miR‐19b) and which is its significance for dopaminergic neurons’ biology remains to be established.

Analysis of PD transcriptome data shows a striking lack of consensus at the level of differentially expressed genes across all tissue sources; however, despite intra‐ and inter‐experimental cell population's heterogeneity, the concordance improves when transcriptome comparisons focus on signalling pathways. Proteasome activity has been reported being altered in transcriptome studies of nigral and extra‐nigral brain regions, blood, or CSF, suggesting a systemic alteration of this pathway in PD.^47^ The proteasome is essential for neuronal survival, and a reduction of the proteasome activity has been correlated with brain ageing and neurodegenerative diseases.^48^ Furthermore, a large body of evidence shows that Dopamine can induce neural cell death, an effect observed in a dose‐ and time‐dependent manner and associated with alteration of proteasome activity.^49^, ^50^, ^51^, ^52^, ^53^ Although restricted to transcript level analysis, our data suggest that this alteration might actually reflect the response to LevoDopa therapy through modulation of miR‐19b levels.

The relationship between miRs and UDP has been explored in multiple in vivo and ex vivo experimental and clinical setups, including the heart, osteosarcoma cells and neurodegenerative disease.^54^, ^55^ Various components of the UDP system were described as miR targets: for example E2 isoforms UE2A, UBE2B, UBE2D3 and UBCH10 are targeted by miR‐7, miR‐455‐5p, miR‐21‐5p and miR‐631, respectively.^56^, ^57^, ^58^, ^59^ The E3‐ubiquitin ligase Parkin associated with familial PD is regulated by miR‐181a in ageing muscles, miR‐103a‐3p levels are increased in an in vitro and in vivo MPTP model of Parkinson's disease, miR‐146a levels are up‐regulated in a rotenone neurodegeneration model, and miR‐218 expression is increased in HEK293 cells.^60^, ^61^, ^62^, ^63^, ^64^ It would be interesting to investigate whether the proposed miR‐19b impact on UDP transcripts translates into an altered neuronal proteasome activity through coordinated alterations of the activity of E2 Ubiquitin‐conjugating enzyme, E3 Ubiquitin ligase system, and Cullin‐2, Cullin‐3 and Cullin‐7 complexes. This would pave the way towards a possible anti‐miR‐19b neuroprotective therapy, since buffering the augmentation of miR‐19b might be an efficient way to prevent or at least diminish the LevoDopa‐associated neurotoxicity.

Although we show that the level of mature miR‐19b increases in the mouse hippocampi and the dopaminergic neurons challenged with LevoDopa, the exact source of the plasma miR‐19b in LevoDopa‐treated patients is yet to be determined. Nevertheless, whether validated in larger, more heterogeneous lots, mir‐19b might serve as an important response biomarker in LevoDopa therapy, opening a new avenue in PD clinical research.

## CONFLICT OF INTEREST

The authors declare no conflicts of interest.

## AUTHOR CONTRIBUTIONS

**Aimee Rodica Chis:** Data curation (equal); Formal analysis (equal); Investigation (equal); Validation (equal); Writing‐original draft (equal); Writing‐review & editing (equal). **Alexandra Ioana Moatar:** Data curation (equal); Investigation (equal); Validation (equal). **Cristina Dijmarescu:** Data curation (equal); Project administration (equal); Resources (equal). **Cecilia Rosca:** Data curation (equal); Resources (equal). **Ruxandra Julia Vorovenci:** Data curation (equal); Resources (equal). **Inge Krabbendam:** Investigation (equal); Validation (equal). **Amalia Dolga:** Resources (equal); Supervision (equal); Writing‐review & editing (equal). **Cristina Bejinar:** Data curation (equal); Investigation (equal); Project administration (equal). **Catalin Marian:** Formal analysis (equal); Funding acquisition (supporting); Methodology (equal); Writing‐review & editing (equal). **Ioan Ovidiu Sirbu:** Conceptualization (lead); Formal analysis (lead); Funding acquisition (lead); Methodology (equal); Supervision (equal); Writing‐original draft (lead); Writing‐review & editing (lead). **Mihaela Simu:** Data curation (equal); Resources (equal); Supervision (equal); Writing‐review & editing (equal).

## Supporting information

Figure S1

Figure S2

Table S1

Table S2

Table S3

Table S4

File S1

File S2

## Data Availability

Data sharing not applicable – no new data generated.

## References

[1] Muangpaisan W, Mathews A, Hori H, Seidel D. A systematic review of the worldwide prevalence and incidence of Parkinson's disease. J Med Assoc Thai. 2011;94(6):749‐755. 21696087

[2] Suchowersky O, Reich S, Perlmutter J, Zesiewicz T, Gronseth G, Weiner WJ. Practice parameter: diagnosis and prognosis of new onset Parkinson disease (an evidence‐based review): report of the Quality Standards Subcommittee of the American Academy of Neurology. Neurology. 2006;66(7):968‐975. 16606907 10.1212/01.wnl.0000215437.80053.d0

[3] Oosterveld LP, Allen JC Jr, Reinoso G, et al. Prognostic factors for early mortality in Parkinson's disease. Parkinsonism Relat Disord. 2015;21(3):226‐230. 25572499 10.1016/j.parkreldis.2014.12.011

[4] Parkkinen L, O'Sullivan SS, Kuoppamäki M, et al. Does levodopa accelerate the pathologic process in Parkinson disease brain? Neurology. 2011;77(15):1420‐1426. 21917769 10.1212/WNL.0b013e318232ab4c

[5] Segura‐Aguilar J, Paris I, Muñoz P, Ferrari E, Zecca L, Zucca FA. Protective and toxic roles of dopamine in Parkinson's disease. J Neurochem. 2014;129(6):898‐915. 24548101 10.1111/jnc.12686

[6] Lewis BP, Burge CB, Bartel DP. Conserved seed pairing, often flanked by adenosines, indicates that thousands of human genes are microRNA targets. Cell. 2005;120(1):15‐20. 15652477 10.1016/j.cell.2004.12.035

[7] Weber JA, Baxter DH, Zhang S, et al. The microRNA spectrum in 12 body fluids. Clin Chem. 2010;56(11):1733‐1741. 20847327 10.1373/clinchem.2010.147405 PMC4846276

[8] Botta‐Orfila T, Morató X, Compta Y, et al. Identification of blood serum micro‐RNAs associated with idiopathic and LRRK2 Parkinson's disease. J Neurosci Res. 2014;92(8):1071‐1077. 24648008 10.1002/jnr.23377

[9] Dong H, Wang C, Lu S, et al. A panel of four decreased serum microRNAs as a novel biomarker for early Parkinson's disease. Biomarkers. 2016;21(2):129‐137. 26631297 10.3109/1354750X.2015.1118544

[10] Vallelunga A, Ragusa M, Di Mauro S, et al. Identification of circulating microRNAs for the differential diagnosis of Parkinson's disease and multiple system atrophy. Front Cell Neurosci. 2014;8:156. 24959119 10.3389/fncel.2014.00156 PMC4051126

[11] Soreq L, Bergman H, Israel Z, Soreq H. Overlapping molecular signatures in Parkinson's patients’ leukocytes before and after treatment and in mouse model brain regions. CNS Neurol Disord Drug Targets. 2013;12(8):1086‐1093. 24040822

[12] Serafin A, Foco L, Zanigni S, et al. Overexpression of blood microRNAs 103a, 30b, and 29a in L‐dopa‐treated patients with PD. Neurology. 2015;84(7):645‐653. 25596505 10.1212/WNL.0000000000001258

[13] Gui Y, Liu H, Zhang L, Lv W, Hu X. Altered microRNA profiles in cerebrospinal fluid exosome in Parkinson disease and Alzheimer disease. Oncotarget. 2015;6(35):37043‐37053. 26497684 10.18632/oncotarget.6158 PMC4741914

[14] Burgos K, Malenica I, Metpally R, et al. Profiles of extracellular miRNA in cerebrospinal fluid and serum from patients with Alzheimer's and Parkinson's diseases correlate with disease status and features of pathology. PLoS One. 2014;9(5):e94839. 24797360 10.1371/journal.pone.0094839 PMC4010405

[15] Ravanidis S, Bougea A, Papagiannakis N, et al. Validation of differentially expressed brain‐enriched microRNAs in the plasma of PD patients. Ann Clin Transl Neurol. 2020;7(9):1594‐1607. 32860338 10.1002/acn3.51146 PMC7480914

[16] Yang Z, Li T, Cui Y, et al. Elevated plasma microRNA‐105‐5p level in patients with idiopathic Parkinson's disease: a potential disease biomarker. Front Neurosci. 2019;13:218. 30936821 10.3389/fnins.2019.00218 PMC6431626

[17] Khoo SK, Petillo D, Kang UJ, et al. Plasma‐based circulating MicroRNA biomarkers for Parkinson's disease. J Parkinsons Dis. 2012;2(4):321‐331. 23938262 10.3233/JPD-012144

[18] Cardo LF, Coto E, de Mena L, et al. Profile of microRNAs in the plasma of Parkinson's disease patients and healthy controls. J Neurol. 2013;260(5):1420‐1422. 23543376 10.1007/s00415-013-6900-8

[19] Li N, Pan X, Zhang J, et al. Plasma levels of miR‐137 and miR‐124 are associated with Parkinson's disease but not with Parkinson's disease with depression. Neurol Sci. 2017;38(5):761‐767. 28181066 10.1007/s10072-017-2841-9

[20] Schwienbacher C, Foco L, Picard A, et al. Plasma and white blood cells show different miRNA expression profiles in Parkinson's disease. J Mol Neurosci. 2017;62(2):244‐254. 28540642 10.1007/s12031-017-0926-9

[21] Ding H, Huang Z, Chen M, et al. Identification of a panel of five serum miRNAs as a biomarker for Parkinson's disease. Parkinsonism Relat Disord. 2016;22:68‐73. 26631952 10.1016/j.parkreldis.2015.11.014

[22] Ma W, Li Y, Wang C, Xu F, Wang M, Liu Y. Serum miR‐221 serves as a biomarker for Parkinson's disease. Cell Biochem Funct. 2016;34(7):511‐515. 27748571 10.1002/cbf.3224

[23] Martins M, Rosa A, Guedes LC, et al. Convergence of miRNA expression profiling, α‐synuclein interacton and GWAS in Parkinson's disease. PLoS One. 2011;6(10):e25443. 22003392 10.1371/journal.pone.0025443 PMC3189215

[24] Ravanidis S, Bougea A, Papagiannakis N, et al. Circulating brain‐enriched MicroRNAs for detection and discrimination of idiopathic and genetic Parkinson's disease. Mov Disord. 2020;35(3):457‐467. 31799764 10.1002/mds.27928

[25] Postuma RB, Berg D, Stern M, et al. MDS clinical diagnostic criteria for Parkinson's disease. Mov Disord. 2015;30(12):1591‐1601. 26474316 10.1002/mds.26424

[26] Kroh EM, Parkin RK, Mitchell PS, Tewari M. Analysis of circulating microRNA biomarkers in plasma and serum using quantitative reverse transcription‐PCR (qRT‐PCR). Methods. 2010;50(4):298‐301. 20146939 10.1016/j.ymeth.2010.01.032 PMC4186708

[27] Peltier HJ, Latham GJ. Normalization of microRNA expression levels in quantitative RT‐PCR assays: identification of suitable reference RNA targets in normal and cancerous human solid tissues. RNA. 2008;14(5):844‐852. 18375788 10.1261/rna.939908 PMC2327352

[28] Heiman M, Heilbut A, Francardo V, et al. Molecular adaptations of striatal spiny projection neurons during levodopa‐induced dyskinesia. Proc Natl Acad Sci USA. 2014;111(12):4578‐4583. 24599591 10.1073/pnas.1401819111 PMC3970487

[29] DennisGJr, ShermanBT, HosackDA, et al. DAVID: database for annotation, visualization, and integrated discovery. Genome Biol. 2003;4(5):P3.12734009

[30] Alieva A, Filatova EV, Karabanov AV, et al. miRNA expression is highly sensitive to a drug therapy in Parkinson's disease. Parkinsonism Relat Disord. 2015;21(1):72‐74. 25465742 10.1016/j.parkreldis.2014.10.018

[31] Sticht C, De La Torre C, Parveen A, Gretz N. miRWalk: an online resource for prediction of microRNA binding sites. PLoS One. 2018;13(10):e0206239. 30335862 10.1371/journal.pone.0206239 PMC6193719

[32] Schulz J, Takousis P, Wohlers I, et al. Meta‐analyses identify differentially expressed microRNAs in Parkinson's disease. Ann Neurol. 2019;85(6):835‐851. 30990912 10.1002/ana.25490

[33] Sourvinou IS, Markou A, Lianidou ES. Quantification of circulating miRNAs in plasma: effect of preanalytical and analytical parameters on their isolation and stability. J Mol Diagn. 2013;15(6):827‐834. 23988620 10.1016/j.jmoldx.2013.07.005

[34] Wang K, Yuan Y, Cho JH, McClarty S, Baxter D, Galas DJ. Comparing the MicroRNA spectrum between serum and plasma. PLoS One. 2012;7(7):e41561. 22859996 10.1371/journal.pone.0041561 PMC3409228

[35] Fernández‐Santiago R, Iranzo A, Gaig C, et al. MicroRNA association with synucleinopathy conversion in rapid eye movement behavior disorder. Ann Neurol. 2015;77(5):895‐901. 25675938 10.1002/ana.24384

[36] Cao XY, Lu JM, Zhao ZQ, et al. MicroRNA biomarkers of Parkinson's disease in serum exosome‐like microvesicles. Neurosci Lett. 2017;644:94‐99. 28223160 10.1016/j.neulet.2017.02.045

[37] Chi J, Xie Q, Jia J, et al. Integrated analysis and identification of novel biomarkers in Parkinson's disease. Front Aging Neurosci. 2018;10:178. 29967579 10.3389/fnagi.2018.00178 PMC6016006

[38] Marques TM, Kuiperij HB, Bruinsma IB, et al. MicroRNAs in cerebrospinal fluid as potential biomarkers for Parkinson's disease and multiple system atrophy. Mol Neurobiol. 2017;54(10):7736‐7745. 27844283 10.1007/s12035-016-0253-0 PMC5684261

[39] Uwatoko H, Hama Y, Iwata IT, et al. Identification of plasma microRNA expression changes in multiple system atrophy and Parkinson's disease. Mol Brain. 2019;12(1):49. 31088501 10.1186/s13041-019-0471-2 PMC6518614

[40] Nie C, Sun Y, Zhen H, et al. Differential expression of plasma exo‐miRNA in neurodegenerative diseases by next‐generation sequencing. Front Neurosci. 2020;14:438. 32457573 10.3389/fnins.2020.00438 PMC7227778

[41] Caggiu E, Paulus K, Mameli G, Arru G, Sechi GP, Sechi LA. Differential expression of miRNA 155 and miRNA 146a in Parkinson's disease patients. eNeurologicalSci. 2018;13:1‐4. 30255159 10.1016/j.ensci.2018.09.002 PMC6149197

[42] Simunovic F, Yi M, Wang Y, Stephens R, Sonntag KC. Evidence for gender‐specific transcriptional profiles of nigral dopamine neurons in Parkinson disease. PLoS One. 2010;5(1):e8856. 20111594 10.1371/journal.pone.0008856 PMC2810324

[43] Cantuti‐Castelvetri I, Keller‐McGandy C, Bouzou B, et al. Effects of gender on nigral gene expression and parkinson disease. Neurobiol Dis. 2007;26(3):606‐614. 17412603 10.1016/j.nbd.2007.02.009 PMC2435483

[44] Lee J, Pinares‐Garcia P, Loke H, Ham S, Vilain E, Harley VR. Sex‐specific neuroprotection by inhibition of the Y‐chromosome gene, SRY, in experimental Parkinson's disease. Proc Natl Acad Sci USA. 2019;116(33):16577‐16582. 31371505 10.1073/pnas.1900406116 PMC6697880

[45] Ameling S, Kacprowski T, Chilukoti RK, et al. Associations of circulating plasma microRNAs with age, body mass index and sex in a population‐based study. BMC Med Genomics. 2015;8:61. 26462558 10.1186/s12920-015-0136-7 PMC4604724

[46] Chaulk SG, Thede GL, Kent OA, et al. Role of pri‐miRNA tertiary structure in miR‐17~92 miRNA biogenesis. RNA Biol. 2011;8(6):1105‐1114. 21955497 10.4161/rna.8.6.17410

[47] Borrageiro G, Haylett W, Seedat S, Kuivaniemi H, Bardien S. A review of genome‐wide transcriptomics studies in Parkinson's disease. Eur J Neurosci. 2018;47(1):1‐16. 29068110 10.1111/ejn.13760

[48] Saez I, Vilchez D. The mechanistic links between proteasome activity, aging and age‐related diseases. Curr Genomics. 2014;15(1):38‐51. 24653662 10.2174/138920291501140306113344 PMC3958958

[49] Stokes AH, Hastings TG, Vrana KE. Cytotoxic and genotoxic potential of dopamine. J Neurosci Res. 1999;55(6):659‐665. 10220107 10.1002/(SICI)1097-4547(19990315)55:6<659::AID-JNR1>3.0.CO;2-C

[50] Michel PP, Hefti F. Toxicity of 6‐hydroxydopamine and dopamine for dopaminergic neurons in culture. J Neurosci Res. 1990;26(4):428‐435. 1977925 10.1002/jnr.490260405

[51] Keller JN, Huang FF, Dimayuga ER, Maragos WF. Dopamine induces proteasome inhibition in neural PC12 cell line. Free Radic Biol Med. 2000;29(10):1037‐1042. 11084292 10.1016/s0891-5849(00)00412-3

[52] Ganguly U, Ganguly A, Sen O, et al. Dopamine cytotoxicity on SH‐SY5Y cells: involvement of α‐Synuclein and relevance in the neurodegeneration of sporadic Parkinson's disease. Neurotox Res. 2019;35(4):898‐907. 30806984 10.1007/s12640-019-0001-0

[53] Ding Q, Keller JN. Proteasome inhibition in oxidative stress neurotoxicity: implications for heat shock proteins. J Neurochem. 2001;77(4):1010‐1017. 11359866 10.1046/j.1471-4159.2001.00302.x

[54] Wei L, Zhang Y, Qi X, Sun X, Li Y, Xu Y. Ubiquitin‐proteasomes are the dominant mediators of the regulatory effect of microRNA‐1 on cardiac remodeling after myocardial infarction. Int J Mol Med. 2019;44(5):1899‐1907. 31485642 10.3892/ijmm.2019.4330 PMC6777676

[55] Zhang Q, Yin X, Zhang Y. MicroRNA‐221 promotes cell proliferation and inhibits apoptosis in osteosarcoma cells by directly targeting FBXW11 and regulating Wnt signaling. Arch Med Res. 2021;52(2):191‐199. 33131925 10.1016/j.arcmed.2020.10.017

[56] Zhao Y, Alexandrov PN, Jaber V, Lukiw WJ. Deficiency in the ubiquitin conjugating enzyme UBE2A in Alzheimer's disease (AD) is linked to deficits in a natural circular miRNA‐7 sponge (circRNA; ciRS‐7). Genes (Basel). 2016;7(12):116. 10.3390/genes7120116 PMC5192492 27929395

[57] Cheng CM, Shiah SG, Huang CC, Hsiao JR, Chang JY. Up‐regulation of miR‐455‐5p by the TGF‐β‐SMAD signalling axis promotes the proliferation of oral squamous cancer cells by targeting UBE2B. J Pathol. 2016;240(1):38‐49. 27235675 10.1002/path.4752

[58] Chang JT, Wang F, Chapin W, Huang RS. Identification of MicroRNAs as breast cancer prognosis markers through the cancer genome atlas. PLoS One. 2016;11(12):e0168284. 27959953 10.1371/journal.pone.0168284 PMC5154569

[59] Xi H, Li L, Du J, et al. hsa‐miR‐631 resensitizes bortezomib‐resistant multiple myeloma cell lines by inhibiting UbcH10. Oncol Rep. 2017;37(2):961‐968. 28000886 10.3892/or.2016.5318

[60] Shimura H, Hattori N, Kubo S, et al. Familial Parkinson disease gene product, parkin, is a ubiquitin‐protein ligase. Nat Genet. 2000;25(3):302‐305. 10888878 10.1038/77060

[61] Goljanek‐Whysall K, Soriano‐Arroquia A, McCormick R, Chinda C, McDonagh B. miR‐181a regulates p62/SQSTM1, parkin, and protein DJ‐1 promoting mitochondrial dynamics in skeletal muscle aging. Aging Cell. 2020;19(4):e13140. 32291905 10.1111/acel.13140 PMC7189996

[62] Zhou J, Zhao Y, Li Z, et al. miR‐103a‐3p regulates mitophagy in Parkinson's disease through Parkin/Ambra1 signaling. Pharmacol Res. 2020;160:105197. 32942015 10.1016/j.phrs.2020.105197

[63] Jauhari A, Singh T, Mishra S, Shankar J, Yadav S. Coordinated action of miR‐146a and Parkin gene regulate rotenone‐induced neurodegeneration. Toxicol Sci. 2020;176(2):433‐445. 32392329 10.1093/toxsci/kfaa066

[64] Di Rita A, Maiorino T, Bruqi K, Volpicelli F, Bellenchi GC, Strappazzon F. miR‐218 inhibits mitochondrial clearance by targeting PRKN E3 ubiquitin ligase. Int J Mol Sci. 2020;21(1):355. 10.3390/ijms21010355 PMC6981953 31948106

