# Supplementary material for: Plasma hsa‐mir‐19b is a potential LevoDopa therapy marker
Source: J Cell Mol Med. 2021 Jul 30;25(18):8715–24. doi: 10.1111/jcmm.16827 (PMC8435426; doi:10.1111/jcmm.16827)

**Figure S1.** Alignment of mir-19b-1 stem-loop sequences of *Canis familiaris*, *Homo sapiens*, *Macaca mulatta*, *Mus musculus*, *Pongo pygmaeus,* and *Rattus norvegicus* showing high conservation of miR-19b-3p (yellow).


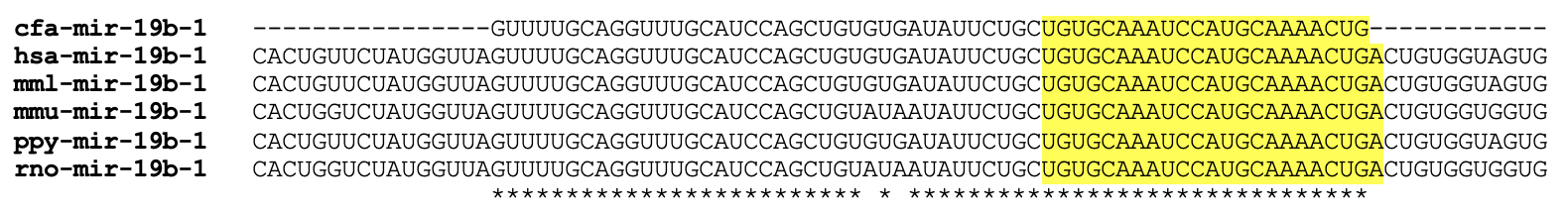

Supplement: Supplementary file 1 — Figure S1 [file JCMM-25-8715-s003.docx]
