# Supplementary material for: Plasma hsa‐mir‐19b is a potential LevoDopa therapy marker
Source: J Cell Mol Med. 2021 Jul 30;25(18):8715–24. doi: 10.1111/jcmm.16827 (PMC8435426; doi:10.1111/jcmm.16827)

**Figure S2.** KEGG representation of Ubiquitin dependent proteolysis (UDP) pathway, with miR-19b targets highlighted in red.


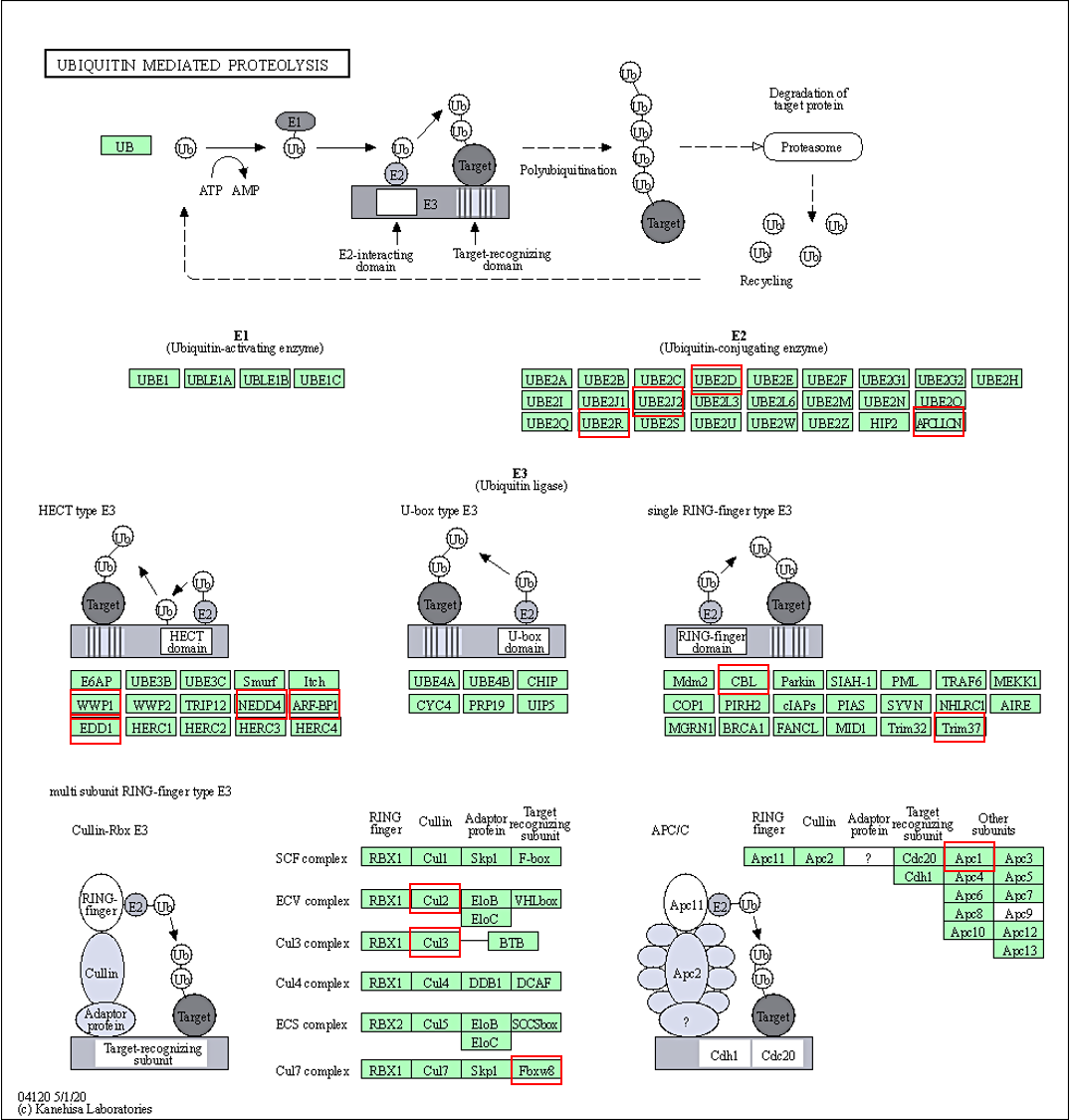

Supplement: Supplementary file 2 — Figure S2 [file JCMM-25-8715-s002.docx]
