# Supplementary material for: Plasma hsa‐mir‐19b is a potential LevoDopa therapy marker
Source: J Cell Mol Med. 2021 Jul 30;25(18):8715–24. doi: 10.1111/jcmm.16827 (PMC8435426; doi:10.1111/jcmm.16827)
Supplement: Supplementary file 3 — Table S1 [file JCMM-25-8715-s007.docx]

**Table S1**

Gender stratification of differentially expressed miRs in PD and controls plasma.

| **Fold change (P value)** | | **miR-16** | **miR-19b** | **miR-19a** | **miR-92a** | **miR-195** |
| --- | --- | --- | --- | --- | --- | --- |
| **Males vs Females** | **Parkinson** | 1.00 (0.998^b^) | 0.698  (0.0192^b^) | 0.737  (0.0292^b^) | 0.608  (0.0014^b^) | 0.973  0.6482^b^) |
|  | **Control** | 0.608  (0.0069^b^) | 0.729  (0.0621^b^) | 0.692  (0.0556^a^) | 0.469  (0.0028^b^) | 0.757  (0.3258^b^) |

^a^ Two-tailed Mann-Whitney test; ^b^ Unpaired t test with Welch's correction
