# Supplementary material for: Plasma hsa‐mir‐19b is a potential LevoDopa therapy marker
Source: J Cell Mol Med. 2021 Jul 30;25(18):8715–24. doi: 10.1111/jcmm.16827 (PMC8435426; doi:10.1111/jcmm.16827)
Supplement: Supplementary file 4 — Table S2 [file JCMM-25-8715-s004.docx]

**Table S2.** Association of normalized Ct values with age and H&Y stage of the disease

| **Pearson r (P-value)** | | **miR-16** | **miR-19b** | **miR-19a** | **miR-92a** | **miR-195** |
| --- | --- | --- | --- | --- | --- | --- |
| **Parkinson** | **Age** | -4.37e-005  (0.999) | 0.092  (0.468) | 0.107  (0.396) | 0.124  (0.327) | -0.047  (0.709) |
|  | **H&Y stage** | 0.066  (0.630) | -0.039  (0.776) | 0.024  (0.862) | 0.152  (0.263) | -0.048  (0.726) |
| **Control** | **Age** | -0.255  (0.182) | -0.279  (0.143) | -0.320  (0.091) | -0.326  (0.085) | -0.220  (0.250) |
