# Supplementary material for: Plasma hsa‐mir‐19b is a potential LevoDopa therapy marker
Source: J Cell Mol Med. 2021 Jul 30;25(18):8715–24. doi: 10.1111/jcmm.16827 (PMC8435426; doi:10.1111/jcmm.16827)
Supplement: Supplementary file 5 — Table S3 [file JCMM-25-8715-s008.docx]

**Table S3.** AUC analyses of miR

|  | **AUC** | **Std Error** | **95% confidence interval** | **P value** |
| --- | --- | --- | --- | --- |
| **miR-16** | 0.643 | 0.058 | 0.529 – 0.757 | 0.0268 |
| **miR-19b** | 0.707 | 0.0576 | 0.594 – 0.819 | 0.0014 |
| **miR-19a** | 0.718 | 0.058 | 0.604 – 0.8315 | 0.0008 |
| **miR-92a** | 0.663 | 0.0617 | 0.5420 - 0.7840 | 0.0117 |
| **miR-195** | 0.7412 | 0.0536 | 0.6093 – 0.8192 | 0.0009 |
