# Supplementary material for: Plasma hsa‐mir‐19b is a potential LevoDopa therapy marker
Source: J Cell Mol Med. 2021 Jul 30;25(18):8715–24. doi: 10.1111/jcmm.16827 (PMC8435426; doi:10.1111/jcmm.16827)
Supplement: Supplementary file 6 — Table S4 [file JCMM-25-8715-s006.docx]

**Table S4.** Two-way ANOVA (α=0.05) analysis (Dunnett's multiple comparisons test) of miR response to Levodopa exposure of dopaminergic neurons

| **Fold change (p value)** | **Levo10** | **Levo20** | **Levo50** |
| --- | --- | --- | --- |
| **miR-19a** | 4.45 (0.0007) | 2.17 (0.0350) | 1.63 (0.1993) |
| **miR-19b** | 3.59 (0.4680) | 15.94 (0.0422) | 51.16 (0.0086) |
| **miR-195** | 1.68 (0.1262) | 2.05 (0.0339) | 1.24  (0.6854) |
